# Lin28 is a critical factor in the function and aging of *Drosophila* testis stem cell niche

**DOI:** 10.18632/aging.101765

**Published:** 2019-02-04

**Authors:** Perinthottathil Sreejith, Wijeong Jang, Van To, Yong Hun Jo, Benoit Biteau, Changsoo Kim

**Affiliations:** 1 School of Biological Sciences and Technology, Chonnam National University, Gwangju 61186, Republic of Korea; 2 Department of Biomedical Genetics, University of Rochester Medical Center, Rochester, NY 14642, USA; 3 Division of Plant Biotechnology, Institute of Environmentally-Friendly Agriculture (IEFA), College of Agriculture and Life Sciences, Chonnam National University, Gwangju 500-757, Republic of Korea

**Keywords:** *Drosophila*, testis, niche, Lin-28, stem cells

## Abstract

Age-related decline in stem cell function is observed in many tissues from invertebrates to humans. While cell intrinsic alterations impair stem cells, aging of the stem cell niche also significantly contributes to the loss of tissue homeostasis associated with reduced regenerative capacity. Hub cells, which constitute the stem cell niche in the *Drosophila* testis, exhibit age-associated decline in number and activities, yet underlying mechanisms are not fully understood. Here we show that Lin28, a highly conserved RNA binding protein, is expressed in hub cells and its expression dramatically declines in old testis. *lin28* mutant testes exhibit hub cell loss and defective hub architecture, recapitulating the normal aging process. Importantly, maintained expression of Lin28 prolongs hub integrity and function in aged testes, suggesting that Lin28 decline is a driver of hub cell aging. Mechanistically, the level of unpaired (*upd)*, a stem cell self-renewal factor, is reduced in *lin28* mutant testis and Lin28 protein directly binds and stabilizes *upd* transcripts, in a let-7 independent manner. Altogether, our results suggest that Lin28 acts to protect *upd* transcripts in hub cells, and reduction of Lin28 in old testis leads to decreased *upd* levels, hub cell aging and loss of the stem cell niche.

## Introduction

Stem cells, which are characterized by their ability to self-renew, present in most adult tissues produce daughter stem cells and differentiated cells. With these unique properties, stem cells replenish aged cells to maintain tissue homeostasis throughout lifespan. With age, most stem cells lose their self-renewing ability, leading to loss of stem cell number, which in turn results in tissue aging and deterioration [1–5]. Importantly, majority of stem cells reside in a specific microenvironment, the stem cell niche, which provides factors responsible for maintenance and differentiation of stem cells in a controlled and coordinated manner. These include local signals that ensure stem cell self-renewal and adhesion of stem cells to their niche compartment. Many evidences suggest that as niches age, their function deteriorates, causing age-related defects in the associated stem cell populations [2,6–8]. Molecular mechanisms underlying niche aging are still poorly understood.

The *Drosophila* testis has been used as an incisive genetic model to provide insights into the mechanisms underlying aging processes occurring in the male germline stem cells and their niches [9,10]. Hub cells, a rosette of 10-12 post-mitotic cells localized at the anterior end of the testis, are the main components of the niche and actively support germline stem cells (GSCs) self-renewal [11]. To this end, hub cells produce DE-cadherin, which mediates adhesion of stem cells to the niche, and secrete self-renewal signaling molecules that are required for stemness, such as unpaired *(*Upd*)* [11]. With age, *Drosophila* testis becomes slender, hub cell number decreases, and GSCs lose their ability to divide [1,6]. Both DE-cadherin and Upd are reduced in aging hub cells, which underlies stem cell loss from the niche [6]. In addition, the expression of IGF-II messenger RNA binding protein (IMP), which stabilizes *upd* mRNA, also declines with age in the *Drosophila* testis [12]. This age-related decline of IMP in the hub cells is caused by the gradual induction of the microRNA Let-7 in aging hub cells, which targets *Imp* RNA for degradation and results in *upd* reduction [12,13].

Lin28 is a conserved RNA-binding protein in higher eukaryotes with function in development, metabolism, differentiation and pluripotency. The best characterized function of Lin28 protein is to act as an inhibitor of the biogenesis of *let-7* microRNAs to reduce mature Let-7 [14–16]. Alternatively, Lin28 acts as a regulator of mRNA stability and translation, by potentially binding thousands of mRNAs [17–26]. Using either one of these properties, Lin28 regulates diverse physiological processes [22,27]. Lin28, for example, functions as a heterochronic factor that regulates developmental timing in *C. elegans* [28]. Lin28 regulates early stage of development in *C. elegans* and Lin28 level is reduced as developmental process progresses [29,30]. In mammals, Lin28 plays a role in cell fate succession, specifying early cell fate, which is analogous to the heterochronic function originally revealed in *C. elegans* [20]. Although much progress has been made on the role of Lin28, particularly in developmental processes as an early cell fate regulator, little is known about the role of Lin28 in the aging process of tissues maintained by a resident stem cell population. Here we show that *Drosophila* Lin28 is specifically expressed in the testis stem cell niche and that its expression dramatically declines with age. Our results reveal a *let-7* independent role of Lin28 in hub cells: Lin28 can directly bind and protect the *upd* mRNA. We finally show that maintaining Lin28 expression in old hub cells prevent the age-related decrease in Upd levels and decline in the niche function, strongly supporting the notion that decline in Lin28 protein in the old niche significantly contributes to the aging process of the *Drosophila* testis.

## RESULTS

### Lin28 is expressed in hub cells

**Figure 1:**
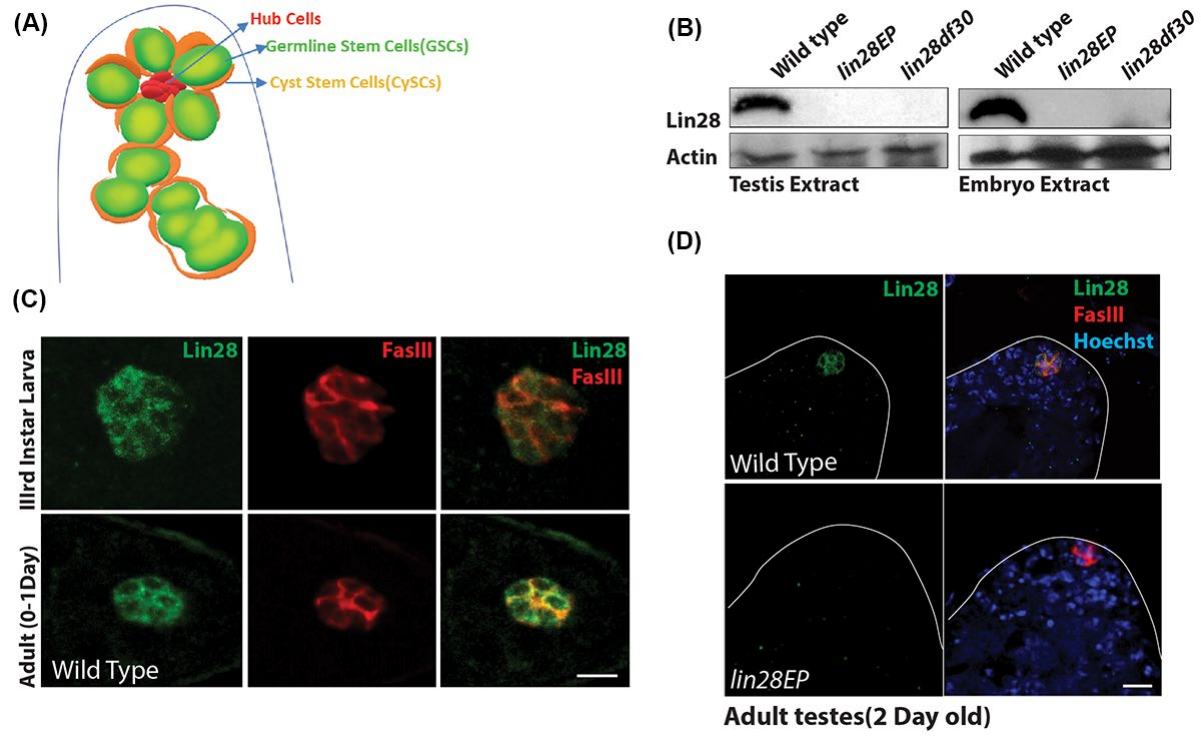
**Expression of Lin28 in *Drosophila* testis.** (**A**) A cartoon depicting the *Drosophila* testis tip, showing the location of hub cells, germline stem cells (GSCs) and cyst stem cells (CySCs). (**B**) Western blot showing levels of Lin28 in testis (left) and embryo extracts (right). Lin28 protein is not detectable in *lin28* mutants. (**C**) Comparison of expression of Lin28 in 3rd larva (up) and young 0-1 Day old adult testis (down). Lin28 is specific to the hub cells. Testis were stained with antibodies specific to Lin28 (green) and hub cell marker FasIII (red). Scale 10µm. (**D**) Adult testis stained with antibodies specific to Lin28 (green), FasIII (red) and Hoechst (blue) in wild-type (top) and *lin28* mutant (bottom), notice Lin28 expression is abolished in the *lin28* mutant.

At the anterior tip of the *Drosophila* testis, hub cells assemble to constitute the niche that supports two stem cell populations, germline stem cells (GSCs) and somatic cyst stem cells (CySCs), with each GSC surrounded by two CySCs (Fig. 1A) [31,32]. To examine the expression of Lin28 in the testis, we generated an antibody directed against the Lin28 protein. We first confirmed the specificity of our antibody using two Lin28 mutant alleles: the insertion line *lin28^EP915^* (*lin28EP*), with a P-element insertion in the 4th exon, and the deletion line *lin28df30,* which deletes several coding exons [27] (Fig. S1A). Western blot analysis showed that Lin28 protein, which is detectable in wild-type testis and embryo extracts, is not detectable in those of both *lin28EP* and *lin28df30* homozygous animals (Fig. 1B), showing that Lin28 is expressed in the fly testis and these two *lin28* mutant alleles are null with regard to protein level.

We next performed co-staining of the testis with an antibody against Fascillin-III protein (FasIII), a hub cell marker. We found that Lin28 is specifically expressed in the hub cells of the stem cell niche in the 3^rd^ instar larva and young adult testis, while it is not expressed in other somatic and germline cells in this organ (Fig 1C, D; Fig. S1C). Importantly, no signal was detected in the hub cells in both *lin28EP* and *lin28df30* homozygotes (Fig. 1D and Fig. S1B), confirming the specificity of the detected signal.

### Hub cell defects in *lin28* mutants

**Figure 2:**
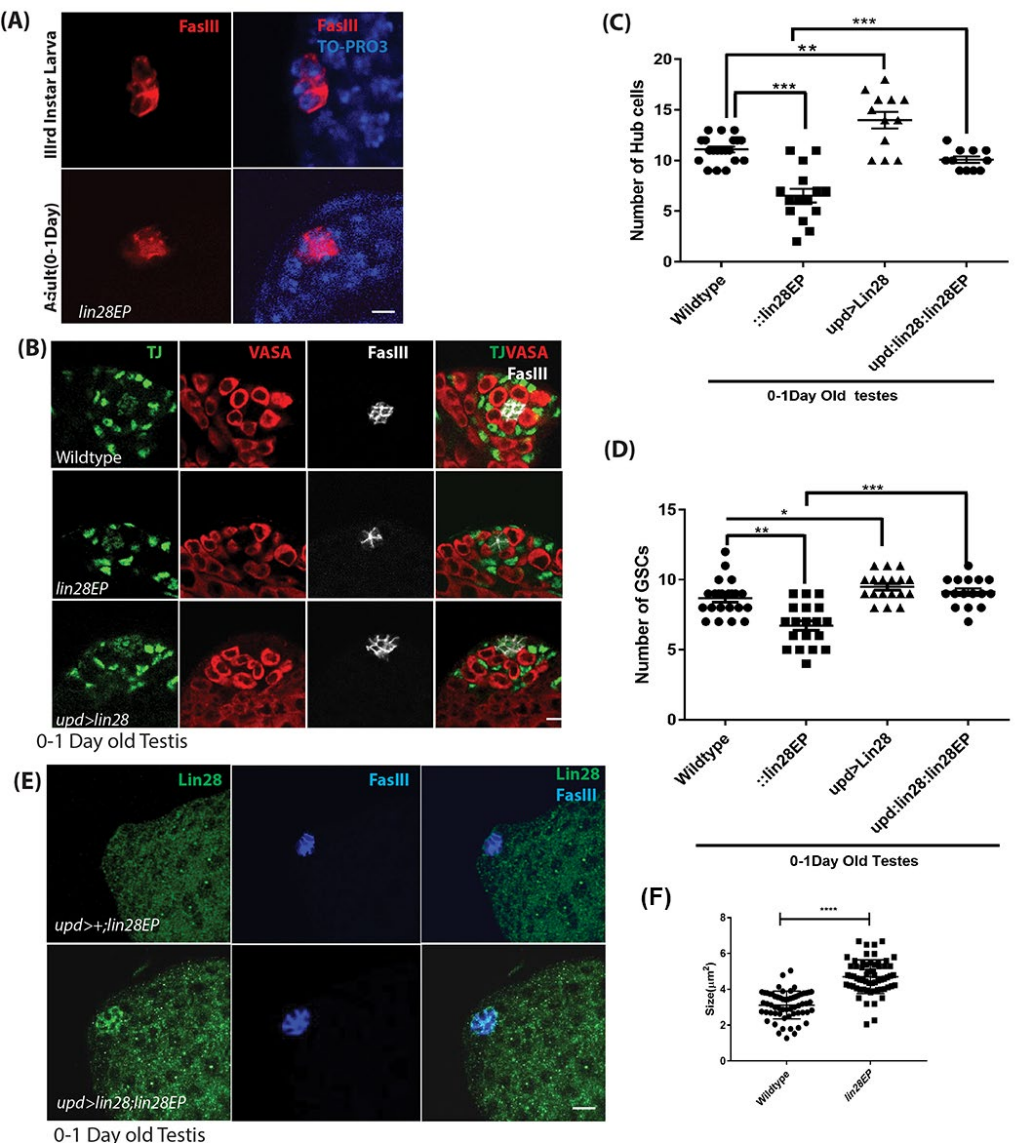
**Phenotypes of *lin28* mutants and overexpression of Lin28.** (**A**) Comparison of the hub cells in 3rd larva (top) and young adult testis (bottom) stained with FasIII (red) and TO-PRO3 (Blue) showing defect in hub cell morphology in *lin28* mutant. Scale 10µm. (**B**) Young adult testis stained with Traffic Jam (TJ), Vasa and FasIII. The number of hub cells is decreased in *lin28* mutant testis. Hub cells are increased in Lin28 overexpressed testis. Scale 10µm. (**C**) Graph depicting the number of hub cells in 0-1Day old wildtype, *lin28* mutant and overexpression of Lin28 along with the rescue of mutant testes by hub specific overexpression of Lin28. ** denotes P<0.01, *** denotes P <0.001 (from Table S1). (**D**) Number of GSCs in 0-1-day old wildtype, *lin28* mutant and overexpressed Lin28 testis, along with rescue of mutant testes by hub specific expression of Lin28. *, **, *** denotes P<0.05, P <0.01, P<0.001 (from Table S1). (**E**) Adult rescued testis showing the expression of Lin28 and FasIII. Lin28 was over expressed in *lin28* mutant background (*upd-GAL4/+: UAS-lin28/+: lin28Ep915*/*lin28Ep915*), which could rescue the mutant phenotype of *lin28* mutant testes (see Table S1). (**F**) The significant increase in the cell size in *lin28* mutant, compared to hub cells in wildtype testes. Scale bar 10µm.

Since Lin28 is expressed in the hub cells we looked for any phenotype in *lin28* mutants. To this end, we took advantage of the two *lin28EP* and *lin28df30* alleles, which our western blot and immuno-staining confirmed as null or severely hypomorphic (Fig. 1B, D). Homozygotes for both alleles are viable, normal in size and show no observable morphological defects. While the fertility of the young mutant males is not affected (Fig. S1), we found that *lin28* mutants present several defects in the hub. Young *lin28* mutant have a significantly reduced number of hub cells with altered morphology (Fig. 2A-C). A typical young (0-1day old) *Drosophila* testis niche consists of approximately 10-12 somatic hub cells arranged in a dome like structure at the anterior tip of the testis [32]. Young *lin28* mutant testis showed reduced hub cells number of 6.53 ± 0.2D8 (n=68) compared to 11.1 ± 0.20 (n=52) in young wild-type controls (Fig. 2B, C and Table S1). In addition to reduced number of hub cells, the morphology of the hub is largely aberrant in *lin28* mutants, as shown by the irregular distribution of FasIII protein, which tends to accumulate at the periphery of mutant hubs, and reduced DE-cadherin levels (Fig. 2A, E, S2A-C), along with increased cell size in *lin28* mutants (Fig. 2F). It is well established that hub cells are essential to maintain GSCs and CySCs population [1,6,9]. Compatible with reduced hub cell number and/or activity, *lin28* mutants show a small but significant decrease in GSCs, identified as Vasa-positive cells immediately adjacent to hub cells, and Cyst Cells, identified by Traffic Jam (TJ) expression excluding the hub cell specific signal (Fig. 2B, D and Table S1).

Importantly, morphological defects and reduced number of hub cells and GSCs were rescued by directing Lin28 expression specifically in hub cells in *lin28* mutants, using the Gal4-UAS system and the hub cell-specific driver *upd*-GAL4 (*upd-GAL4: UAS-lin28: lin28EP^EP915^*) (Fig. 2E, Table S1). Altogether, our data demonstrate that the function of Lin28 is required cell-autonomously in hub cells for their proper number and development.

### Lin28 expression decreases with age and maintaining Lin28 expression prevents niche aging

Remarkably, most of the phenotypes observed in *lin28* mutant testes, such as decreased cell number and DE-Cadherin levels, recapitulate changes observed during normal aging [1,6]. Thus, we asked whether Lin28 expression varies with ages and whether such expression changes could contribute to niche aging.

**Figure 3:**
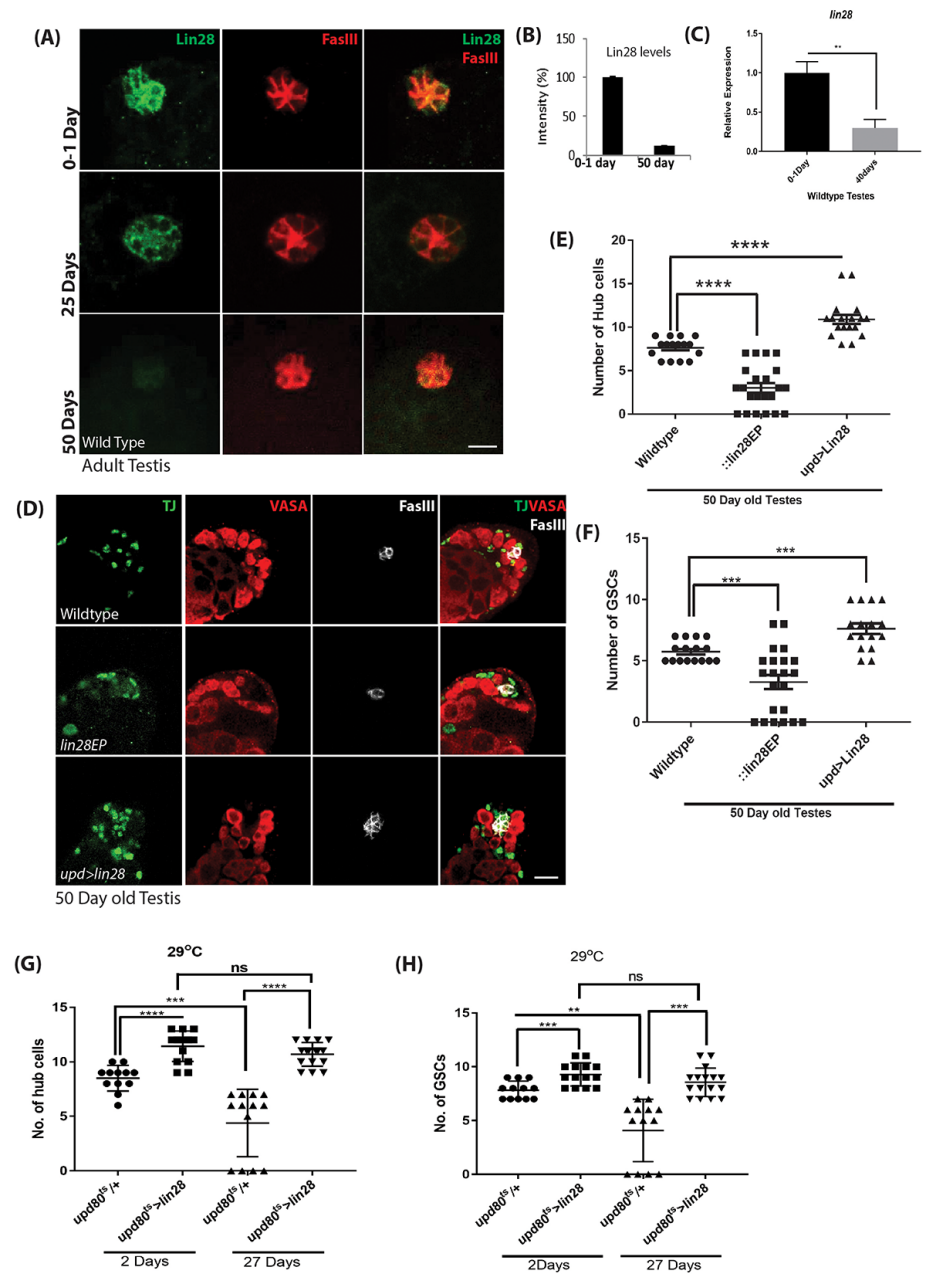
**Age dependent loss of Lin28 in hub cells.** (**A**) Adult testis stained with Lin28 (green) and FasIII (red) in an age dependent manner. Lin28 level, but not FasIII, is decreased in old testis. Scale 10µm. (**B**) Comparison of the level of intensity of green signal (Lin28 expression) in young (0-1 Day) *vs* old testis (50 Day). (**C**) Comparison for the transcript levels of lin28 in young (0-1 Day) and old (40 Days) wildtype testis, showing the reduction in Lin28 levels with age. (**D**) Hub cell number is not decreased in old testis when Lin28 is overexpressed (*upd-Gal4*>*UAS-lin28*) as compared to wild type testis. Testis were stained with Vasa, Traffic Jam (TJ) and FasIII antibodies. Scale 10µm. (**E**) The number of hub cells in old testis is comparatively reduced in *lin28* mutant testis compared to wildtype testis. The number of hub cells in old upd>lin28 (*upd-Gal4*>*UAS-lin28*) testis is similar to a young wild type testis (data from Table S1). (**F**) The number of GSCs in 50-day old wildtype, *lin28* mutant and upd>lin28 (*upd-Gal4*>*UAS-lin28*) testis (data from Table S1). (**G**) Comparison of the number of hub cells in wildtype *vs* adult specific overexpression of Lin28 in an age dependent manner (*upd-GAl4; tubGAL80ts: UAS-lin28*). Overexpression of Lin28 clearly increases the number of hub cells and with age the number of hub cells is maintained when lin28 is maintained compared to loss of hub cell in wildtype testes with age. (**H**) Comparison of the number GSCs in wildtype testes and adult specific over expression of Lin28 in hub cells. The number of GSCs reciprocates the number of cell number. * denotes, P<0.05. ** denotes P<0.01, *** denotes P<0.001, and **** denotes 0.0001.

We found that Lin28 protein levels dramatically decreased in aging animals (Fig. 3A, B), becoming almost undetectable in 50-day-old testes. In addition, at a transcript level Lin28 expression is reduced significantly in the old testes (Fig. 3C). Next, we reasoned *lin28* mutation may exacerbate testis aging phenotype and maintaining Lin28 expression in the hub cells of aging animals may counteract testis aging. To test this hypothesis, we quantified the number of hub cells in old (50-day-old) control males, *lin28* mutants and animals with *lin28* over-expressed specifically in Hub cells (*upd-GAL4>UAS-Lin28*) (Fig. 3D, E and Table S1). In wild-type males, 7.6 ± 0.33 (n=40) hub cells were observed while 3 ± 0.30 (n=60) hub cells were observed in *lin28* mutant (Fig. 3 D, E). 5% of the testis showed complete loss of hub cells in the mutant old testis. This confirmed the previously reported decrease in hub cell number during normal aging [1,6] and suggested that aging is significantly accelerated in *lin28* mutants. Conversely, hub cell specific over-expression of Lin28 significantly increased the number of hub cells from 11.1± 0.20 to 13.6 ± 0.26 (n=42) in young testis and from 7.6 ± 0.33 to 10.8 ± 0.36 (n=38) in 50-day old testis (Fig. 3D, E and Table S1), suggesting that ectopic Lin28 can enhance and maintain stem cell niche function in old testes. As for young testes, changes in hub cell numbers in old animals were mirrored by similar changes in the number of GSCs and apical somatic cyst cells excluding the hub cells (Fig. 3D, F and Table S1).

We show that absence of *lin28* or over-expression in hub cells throughout development results in small but significant changes in hub cells and GSCs number in young animals, raising the possibility that the effects observed in aging tissues results from developmental defects. Thus, to further characterize the adult specific role of Lin28 in the hub cells and its connection to the aging process, we carried out adult-specific Lin28 expression. To this end, we combined the *upd-GAL4* driver with the *tubulin-GAL80ts temperature* sensitive GAL-80 inhibitor (*updG80^ts^*) [33] and analyzed animals grown at permissive temperature until adult and switch to 29^o^C for 2 days (young) or 27 days (old). Our data show that Lin28 over-expression increases the number of hub cells 2 days after transgene induction, (11.43 ± 0.37, n=42) compared to wild type control (8.5 ± 0.33, n=36) (Fig. 3G, S3A, Table S2). More importantly, we found that, while a strong age-related loss of hub cells is observed in control testes, no significant age-dependent change in hub cell number is observed when Lin28 expression is maintained throughout adulthood specifically in hub cells (young (11.43 ± 0.37, n=42) to old testes (10.69 ± 0.26, n=48), Table S2). These changes or absence of changes in hub cell numbers were mimicked by the number of GSCs in the niche (Fig. 3H, S3, Table S2).

Together these data strongly suggest that the aging niche becomes less functional and abnormal because of decreased expression of Lin28 in hub cells and that maintaining Lin28 expression in these cells is a viable approach to prevent testis aging.

### Lin28 regulates Upd expression and STAT signaling in the hub

**Figure 4:**
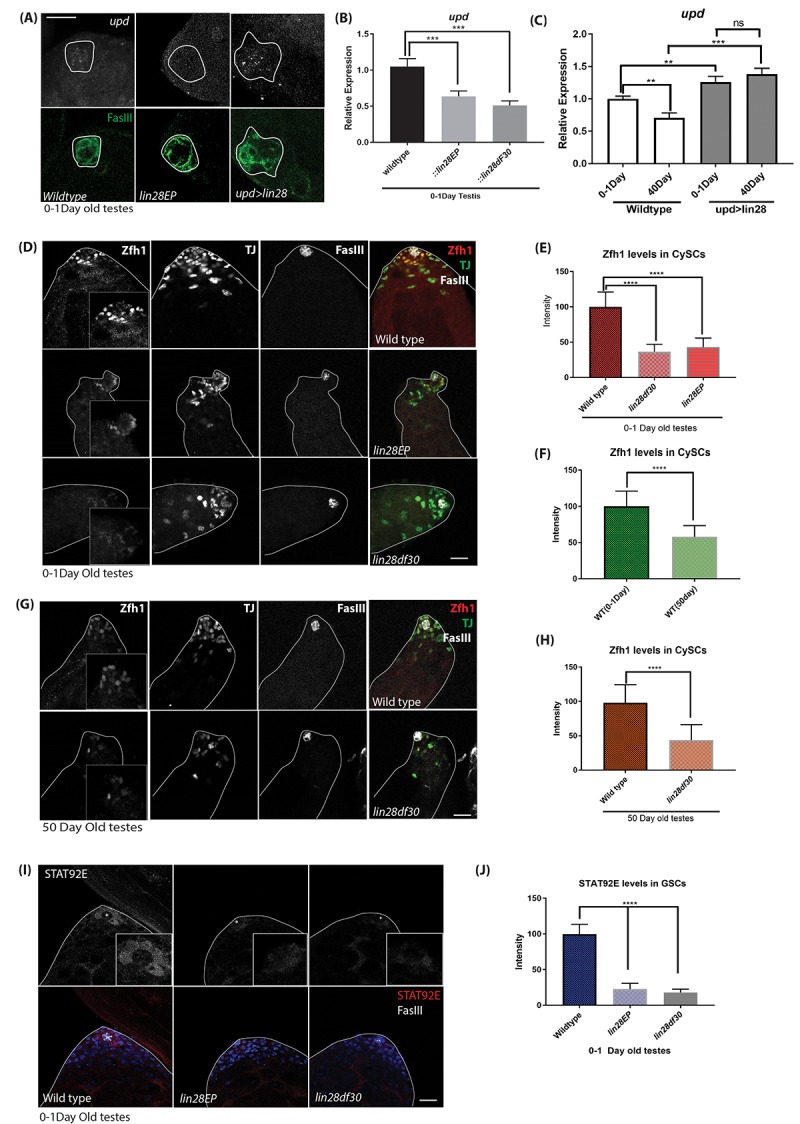
**Upd-JAK-STAT signaling is affected with loss of Lin28.** (**A**) In-situ hybridization of young (0-1 day old) testis. *upd* RNA level is decreased in *lin28* mutant testis relative to wild-type and increase in *upd* levels in hub specific expression of Lin28. Scale 10µm. (**B**) quantitative RT-PCR of young (0-1day old) testis, *upd* mRNA level is decreased in *lin28* mutants relative to wild-type. (**C**) RT-qPCR of young *vs* old testis showing reduced levels of *upd* mRNA in WT testis with age, while, maintained levels of *upd* in overexpression of Lin28 with age. (**D**) Young (0-1-day old) testis stained with Zfh1, TJ and FasIII antibodies. Zfh1 level, but not TJ and FasIII, is reduced in *lin28* mutants. Scale 10µm. (**E**) Quantification of Zfh1 levels in CySCs shows reduced Zfh1 levels in mutants. [wildtype, n= 197; lin28EP, n=220; lin28df30, n= 185]. (**F**) Comparison of Zfh1 levels in young and old WT testes indicating that with age Zfh1 level goes down due to loss in STAT activity. [WT young, n= 197; WT old, n= 145]. (**G**) 50-day old testes stained with Zfh1, TJ and FasIII antibodies showing reduced levels of Zfh1 with age. Scale 10µm. (**H**) Comparison of Zfh1 levels in WT *vs* mutant [ wildtype, n= 145; lin28df30, n=125] (**I**) STAT92E protein level is reduced in the mutants. Scale 10µm. (**J**) Comparison of STAT92E levels in WT (n=115) *vs* mutants (*lin28EP* n=92; *lin28df30*, n=97). STAT92E protein level is reduced in *lin28* mutants; * depicts the hub area. ** denotes P<0.01, *** denotes P<0.001, **** denotes P<0.0001, ns denotes not significant.

Lin28 expression pattern is identical to the expression pattern of the key self-renewal factors Upd and hub cell adhesion protein DE-cadherin, namely that they all are highly expressed in hub cells and show decreased expression with age [6]. This raised the possibility that *upd* and DE-cadherin reduction with age might be due to reduction of Lin28 with age. Therefore, we examined whether the expression of *upd* and DE-cadherin is dependent on Lin28. In-situ hybridization of *upd* transcripts confirmed that *upd* level is reduced in the mutant hub area (Fig. 4A), conversely hub specific expression of Lin28 leads to increased *upd* transcripts in the hub area. Quantitative RT-PCR showed that *upd* (Fig. 4B) and *DE-Cadherin* (Fig. S2C) transcript levels are reduced in *lin28* mutant testes relative to wild-type. Conversely, over-expressing Lin28 in hub cells using *upd-GAL4* significantly increases u*pd* mRNA level (Fig. 4C). More importantly, confirming the role of Lin28 in hub cell aging, we observed an age-related reduction in the mRNA levels of *upd* in wild-type testes, while continued expression of Lin28 in hub cells of old testes maintains the expression of *upd* similar to young testes (Fig. 4C).

Secretion of upd in the hub cells activates the JAK-STAT signaling in the GSCs and CySCs, which is essential for their maintenance [11,34]. Zfh1, a transcriptional repressor is known as a somatic target of JAK-STAT signaling, expressed in CySCs [11,34]. Stabilized STAT92E protein can be used as a read-out for JAK-STAT signaling [35,36] Thus, we used these two markers to confirm that the Upd reduction in *lin28* mutant hub cells affects STAT signaling in surrounding cells. Supporting our model, we found that Zfh1 protein level is reduced in CySCs of *lin28* mutant testis (Fig. 4D, E) and that STAT92E protein is lost in the GSCs of *lin28* mutants (Fig. 4I, J). In contrast, several factors that are expressed in hub cells such as FasIII and TJ protein levels were not altered in *lin28* mutants (Fig. 4D). While with age the expression of Zfh1 goes down (Fig. 4F), we saw that the expression of Zfh1 further goes down in *lin28* mutant testes (Fig. 4G, H).

Previously, it was shown that IMP stabilizes *upd* mRNA from siRNA mediated degradation in hub cells and with age, loss of IMP leads to loss of *upd* transcripts [12]. Age associated loss of IMP is related to post-transcriptional regulation of IMP transcripts by the microRNA let-7. Lin28 is a well-established inhibitor of biogenesis of *let-7* microRNAs [37]. This raised the possibility that Lin28 controls *upd* expression through this *let-7* / IMP axis. However, while we observed a slight reduction of IMP transcripts in young Lin28 mutant testes, we did not detect any reduction in expression of IMP protein in either young or old tissues (Fig. S4A, B, D). Similarly, over-expression of Lin28 in hub cells has limited effect on IMP expression in young or old testes, as measured by RT-qPCR or immunostaining (Fig S4A, C, D). Of note, an accumulation of IMP protein into granular structures, which co-localize with the stress granule marker Cup, in the hub area of the mutant testes can be observed in some samples (Fig. S4A, D, E), suggesting that IMP sub-cellular localization and function may be affected by the loss of Lin28. We next tested whether mature let-7 level is influenced by *lin28* gene dose. We found that mature let-7 level is low in young animals and not significantly different between young wild-type and mutants (Fig. S4F). As reported previously [12], mature let-7 level dramatically increases with age in control tissues (Fig. S4F). However, this is not significantly different when Lin28 is mutated or over-expressed (Fig. S4F, G), suggesting that Lin28 does not regulate let-7 during the hub cell aging process.

Altogether, our results demonstrate that Lin28 controls *upd* expression in hub cells, in a largely *let-7*-independent manner.

### Lin28 binds and stabilizes the *upd* transcript.

Lin28 is a translational regulator that was shown to bind several target mRNAs directly. Our *in vivo* experiments suggested to us that Lin28 may directly control the stability of the *upd* transcript. Thus, we examined the relationship between *upd* transcript level and Lin28 in S2 cells.

**Figure 5:**
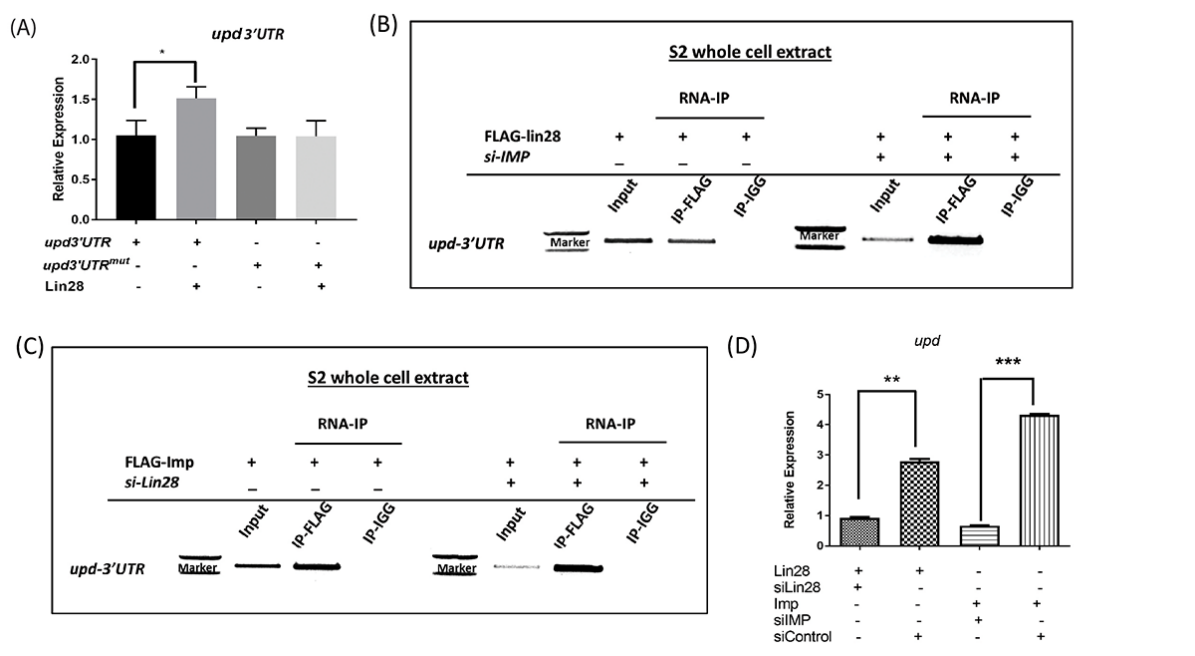
**Lin28 and IMP associate with *upd* RNAs.** (**A**) qRT-PCR showing the relative levels of *Luc-Upd3’UTR* reporters in cells co-transfected in the presence and absence of Lin28, showing increased Luc-Upd reporter expression in presence of *lin28*. Site directed mutagenesis of the Lin28 binding GGAGA motif abolishes the stabilization induced by Lin28 expression. (**B** and **C**) RNA-immunoprecipitation performed on S2 cells transfected with HA-tagged Lin28 and Flag-tagged IMP. Immunoprecipitation followed by RT-PCR detects that IMP and Lin28 immunoprecipitants contain *upd* RNAs. Depletion of either Lin28 or IMP does not affect RNA binding of the other protein. (**D**) Quantification of RNA-immunoprecipitation. siControl: scrambled siRNA. * denotes P<0.05, ** denotes P<0.01, *** denotes P<0.001.

We confirmed that Lin28 over-expression in S2 cells increases the expression of a *upd* reporter which contain an intact 3’UTR, suggesting that the regulatory mechanisms present in hub cells are at least partially conserved in cultured cells (Fig. 5A). To first test whether Lin28 may bind the *upd* transcript directly, we mutated the putative binding site of Lin28 (GGAGA motif) present in *upd*3’UTR at position 228 and found that overexpression of Lin28 has no effect on the expression of this construct (Fig. 5A). We next carried out Lin28 immunoprecipitation followed by RT-PCR for *upd* mRNAs. Lin28 immunoprecipitates from S2 extracts are highly and specifically enriched in *upd* transcripts as compared to control reactions, demonstrating that Lin28 binds *upd* transcripts directly in these cells (Fig. 5B). The IMP protein has also been shown to bind *upd* mRNAs [12]. We confirmed that immunoprecipitating Flag-tagged IMP in S2 cells significantly enriches in *upd* mRNAs (Fig. 5C). We next asked whether these two binding events are dependent on each other. To this end we performed Lin28 immunoprecipitation while knocking-down IMP using siRNA and the converse experiment. Our results show that Lin28 and IMP proteins’ ability to bind to *upd* transcripts is not significantly affected in the absence of the other factor (Fig. 5B, C). Supporting the notion that both Lin28 and IMP can stabilize the *upd* transcript independent of each other, we found that overexpressing Lin28 while knocking down IMP is sufficient to increase *upd* levels and conversely IMP over-expression stabilize *upd* in the absence of Lin28 (Fig. 5D).

Together, our biochemical studies demonstrate that Lin28 is capable of binding to and controlling the stability of the *upd* transcript, suggesting that it may control stem cell niche function in the testis at least in part through the regulation of Upd expression.

## DISCUSSION

### Lin28 is required for niche development and long-term function

Lin28 has been known to function in diverse biological contexts like development, metabolism, pluripotency; along with a role in disease such as cancer [19,37]. In this report, our identification of Lin28 as one of the key factors regulating the male germline stem cell niche function extends our understanding of the diversity of roles Lin28 plays in stem cell biology. The study of the mechanism underlying the regulation of self-renewal factors in the hub cells provides us with new insights into the proper niche function. Our data establishes that Lin28, an RNA binding protein, plays a pivotal role in somatic post-mitotic hub cells of the *Drosophila* testis stem cell niche. We show that hub cell specific expression of Lin28 starts in early stages of testis development, plateaus in young adults and deteriorates with age. Similar to the niche deterioration observed in older testes, Lin28 mutations result in a loss in hub cell number, associated with impaired hub morphology due to aberrant increase in the cell size. Importantly, we show that hub cell specific expression of Lin28 is sufficient to rescue the mutant phenotype and that maintaining the expression of Lin28 in an adult specific manner is sufficient to preserve the number of hub cells in old testes, strongly suggesting a cell-autonomous function of Lin28 in regulation of long-term hub cell number, morphology and function.

While it is unclear how hub cells are lost and why their size increases in absence of Lin28, we attributed these phenotypes to a non-apoptotic mechanism: Tunnel assay and immunohistochemistry against cleaved caspase 3 (an apoptotic marker) did not show any significant change between control and *lin28* mutant testes, suggesting the hub cell death or loss is not due to apoptosis (data not shown). Other mechanisms leading to hub cell loss are possible. For example, we show that E-cadherin accumulation around hub cells is strongly reduced in *lin28* mutants, suggesting that the integrity of the niche is compromised, and hub cells may migrate away and lose their identity. Alternately, hub cells may trans-differentiate into CySCs when Lin28 is over-expressed similar to what was reported in [38], however, this possibility has been challenged by others [39]. Conversely, we show that over-expressing Lin28 throughout development or during adulthood results in a small but significant increase in hub cell number. Our studies have not allowed us to detect any dividing hub cells (positive for the phospho-Histone H3 mitotic marker) in these conditions (data not shown). However, previous studies have shown that germ cells and CySCs can repress hub cell development [39,40] the absence of these cues may trigger higher number of hub cells in Lin28 over-expressing conditions. Further experiments are required to understand whether Lin28 affects these mechanisms controlling specification and development of hub cells and CySCs and whether these signaling interactions between the cell types that compose the testis niche are maintained in the adult. We anticipate that the identification of Lin28 targets in hub cells may provide additional insights into the mechanism(s) by which Lin28 controls hub cell numbers.

### Lin28 controls *upd* expression and regulate niche aging

Our data demonstrate that Lin28 controls the expression of self-renewal factor *upd* in hub cells and that its loss in the hub niche participates to the impaired Upd expression and niche function in older testes. Importantly, our data strongly suggest that Lin28 directly binds to the *upd* mRNA 3’UTR to control its stability in hub cells. In addition, we show that let-7 expression is largely not affected by Lin28 in testis, in particular in old animals where let-7 expression is highly induced and associated with the loss of Upd [12]. Recently, in *Drosophila* it was shown that Lin28 does not affect the biogenesis of let-7 [41] suggesting that the molecular mechanism for Lin28 function can vary. Our work thus identifies a novel mechanism that adds complexity to our previous view of the regulation of Upd expression during aging. In short, Upd is destabilized in old testis by two mechanisms acting in parallel: the increased *let-7* expression which causes IMP loss; and the loss of Lin28 expression which directly affects the *upd* transcript. Why two parallel, although connected, pathways are necessary to maintain *upd* expression remains unclear at this point.

Our cell culture data suggest that both Lin28 and IMP can bind and stabilize the *upd* 3’UTR in the absence of the other factor. Our study indicates that *Drosophila* Lin28, like mammalian Lin28a [24], likely binds to mRNAs target including *upd* at the sequence specific motif (GGAGA). IMP binds to *upd* 3’UTR at a different sequence [12]. Whether single RNA molecule can be bound by both factors at the same time and whether this may further stabilize the target transcripts remains unexplored. It will also be interesting to investigate why both Lin28 and IMP can bind to *upd* mRNA independently and whether binding of Lin28 to *upd* induce any conformational changes which assists binding of IMP to *upd* mRNA or reciprocally. Interestingly in our hands, the absence of one of the 2 factors i.e. Lin28 or IMP, leads to an increase in *upd* mRNA level, suggesting that Lin28 and IMP can protect *upd* mRNA in absence of the other, and may have greater affinity for the *upd* 3’UTR in these conditions. This raises the possibility of Lin28 or IMP working in parallel pathways or at different stages of upd regulation. Whether this mechanism is mirrored in-vivo need to be further characterized. Functionally, it is clear from our studies and others that both factors are required to prevent accelerated aging in hub cells. It was observed that IMP protein accumulates in granular structures specific to the hub area of *lin28* mutants. Preliminary investigation indicates that these foci are stress granules (Fig. S4E), whether this affects IMP function and upd stabilization, needs further characterization.

### Other Lin28 targets in hub cells

Although the decreased number and morphological defects in Lin28 mutant hub cells may be attributed to loss of *upd*, it is likely that other factors are regulated by Lin28 and play a critical role in this phenotype and the overall decreased stem cell niche function. Other factors known to contribute to the niche function such as *DE-cadherin*, *dpp*, *esg*, *Rbf* are prime candidates that will have to be investigated. It is interesting to note that, in a different fly tissue Lin28 binds directly to the Insulin receptor *InR* mRNA in intestinal stem cells and regulate their symmetrical renewal [22]. *InR* function is required cell autonomously for GSC maintenance in *Drosophila* testis [42]; it will thus be interesting to test whether hub cell specific Lin28 has similar function. Further studies are required to understand how Lin28 might regulate other factors or signaling pathways present in the niche.

Age related changes in the stem cell niches have been known to directly influence stem cell function and self-renewal ability [6,8,9]. Age associated decline in stem cells function and niches is known to regulate aging. Understanding the mechanistic basis of stem cell behavior in the niche will lead us to the development of strategies to facilitate stem cell-based therapies. Future work involves broadening our understanding of the mechanism of stem cell renewal with Lin28 as a key regulator.

## MATERIALS AND METHODS

### Fly stocks and husbandry

*lin28* mutant line *w[1118];P*(w[+mC]=EP)*lin-28[EP915]* (#17298), were obtained from Bloomington *Drosophila* Stock Center. *W1118* from Bloomington *Drosophila* Stock Center was used as a wildtype control. *upd-GAL4* was a kind gift from S. Dinardo. *lin28df30* was a kind gift from F. Michon. *UAS-Lin28* was generated in the lab. *upd-GAL4;tub-GAL80ts* was a kind gift from Christian Bokel.

All *updGal4; tubGAL80ts* and *updGal4;tub-GAL80ts>UAS-lin28* flies were raised at 18^o^C unless otherwise noted. Flies were raised at 18^o^C for 5 days post-eclosion to restrict GAL4 during final developmental stages. Flies were shifted to 29^o^C for 2 days to inhibit Gal80ts and activate GAL4 or aged for 27 days at 29^o^C.

### Quantification of Hub cells and Germline Stem cells, and hub cell size

To quantify the number of hub cells, the testis was stained with antibody specific to hub cell, FasIII and TO-PRO3/Hoechst a nuclear marker. Densitometric analysis of Serial Z section was used to quantify the number of hub cells using LAS X software from Leica. The number of GSCs were quantified by the cells positively stained to VASA which were directly in contact to the FasIII positive hub cells. The size of the hub cells was measured as described [43].

### Cloning

The *lin28* cDNA (RE05342) was obtained from *Drosophila* Genetic Resource Centre. RE05342 consists of pFLC-1 vector which is a derivative of pBluescriptII-SK (+) with the cDNA insert (1165bp) of *lin-28* (transcript 1152bp with *5’UTR* and *3’UTR*). The *Not I-Xho I* insert of the *lin28 CDS* (588bp) was sub-cloned into pAc5.1/V5- HisA-HA/FLAG (Invitrogen) vector for S2 cell expression, while *Bgl II- Xho I* insert and *EcoR I-Xho I* insert was sub-cloned into *pUASt* for fly expression studies. The *KpnI-Not1* insert was sub-cloned into phmKGN/C-MC/MN vector for fragment complementation assay. The IGF-II mRNA binding protein (*IMP*) cDNA (RE72930) was obtained from *Drosophila* Genetic Resource Centre. RE72930 consists of derivative of pBluescriptII-SK (+) with cDNA insert (3955bp) of *IMP-RJ* (3942bp transcript with *5’UTR* and *3’UTR*). The *Not I-Xho I* insert of the *IMP* CDS (1743bp) was sub-cloned into pAc5.1/V5-HisA-FLAG for S2 cell related experiments.

The outstretched (os)/unpaired (upd) cDNA (*BS-UPD*) was obtained from Douglas A. Harrison. *BS-UPD* consists of the cDNA of upd (2520bp). The XhoI -Xba I insert of 3’UTR (740bp) of *upd* was sub-cloned into pAc5.1A(Invitrogen) following a luciferase reporter (*pAc5.1A-Luc-upd3’UTR*). Site directed mutagenesis was used to generate *pAc5.1A-Luc- upd3’UTRmut* (^228^GGAGA=AACAT).

### Generation of Lin28 antibody

The N-Terminal peptide sequence Lin28- NGLERRTTSQSSTSSAN was used to generate the antibody against Lin28 in rat by Peptron (Korea).

### Immunostaining

Immunostaining was done as described [44]. Briefly, for third instar larvae, the male larvae were hand dissected in Schneider’s media using micro scissors and micro forceps. Using the micro scissor, the larva was cut into half and the internal organs especially the gut was pulled out from the posterior region carefully, preventing the other organs from losing. This posterior region containing testis was fixed in 4% formaldehyde in PBS for 20minutes, followed by washing in PBX. It was then blocked in 5% Normal Goat serum for 1hour and incubated in appropriate antibody overnight at 4^o^C. Secondary antibodies were used at 1:500. After the secondary antibody treatment, the testis was carefully detached from the posterior region using tungsten needles and mounted in antifade. Adult testis of appropriately aged male flies were immunostained as previously described except they were hand dissected in Ringer’s solution and fixed in 4% formaldehyde for 40 minutes.

The following primary antibodies were used along with the following concentration: Rat anti-Lin28 1:100 (This study), mouse anti-FasIII 1:30, rat DE Cadherin 1:30, rat anti-VASA 1:400 (Developmental Studies Hybridoma Bank, DSHB), Rabbit anti-VASA 1:3000, Rabbit anti-Zfh1(1:1000) (Ruth. Lehmann), guinea pig anti-Traffic Jam 1:10000 (Dorothea Godt), rabbit anti-IMP 1:1000 (Paul MacDonald), Rat anti-Cup 1:1000 (A. Nakamura), Rabbit anti-GFP 1:500 (Invitrogen, USA), Rabbit anti- STAT92E 1:1000 (Denise Montell). The secondary antibodies were used in 1:500 (Alexa Series, Invitrogen, USA).

### RNA-Immunoprecipitation

S2 cells transfected with HA tagged Lin28 and FLAG tagged IMP were washed twice in ice cold PBS and were subjected to RNA-IP. The washed cells were lysed using polysome lysis buffer containing protease inhibitor cocktail, RNase inhibitor and Vanadyl complexes along with DTT. The lysed cells were centrifuged at high speed for 15 minute and frozen for 30 minutes at -80^o^C. The thawed lysate was subjected to Immunoprecipitation using HA-Agarose or FLAG-Agarose beads for 4 hours at 4^o^C and washed 5 times in wash buffer followed by 3 times washing in wash buffer with 1M urea. The washed Agarose beads were subjected to DNAse1 treatment for 10 minutes at 37^o^C followed by proteinase K (10 mg/ml) treatment at 55^o^C for 30 minutes. The proteinase K treated beads were subjected to Phenol: Chloroform: Isoamyl alcohol extraction to extract RNA. The RNA was diluted and was subjected to reverse transcription using reverse transcriptase. The RT samples were used as template to amplify RNA bound to the flagged proteins by PCR.

When using the luciferase-based reporters, S2 cells were transfected with reporter constructs of *Luc-upd3’UTR* or *Luc-upd3’UTR^mut^* using DDAB transfection reagent (Fluka/Sigma). 72 hours after transfection cells were harvested, and total RNA was extracted with Trizol (Invitrogen). Following reverse transcription, qRT-PCR was used to measure *Luc-upd3’UTR* levels using *upd3’UTR*-specific primers with *rp49* as control.

### In-situ hybridization of *upd* RNA

RNA in situ hybridization was performed as previously described [11]. Full length cDNA of UPD cloned into plasmid was linearized to generate probes using the Roche RNA labeling kit (Roche, USA).

### qRT-PCR

Total RNAs were extracted from young (0-1 day old) and old (30-32 days old or specified) testis using mirVana miRNA isolation kit (Invitrogen). Total RNA (200 ng) was used for cDNA synthesis with specific *let-7* stem loop primer 5’-GTCGTATCCAGTGCAGGGTCCGAGGTATTCGCACTGGATACGACACTATA-3’ and *let-7,* forward, 5’-GCCGCTGAGGTAGTAGGTT GTA-3’ and reverse 5’-GTGCAGGGTCCGAGG-3’, as described previously [45]. Quantitative PCR was performed with SYBR green (Applied Biosystems, USA) on a Step One Real-Time PCR Systems from Thermo Scientific according to the manufacturer’s instructions. U6 snRNA (RT primer 5’-CGCTTCACGAATTTGCGTGTCAT-3’ and forward 5’-GCTTCGGCAGCACATATACTAAAAT-3’, reverse 5’-GTGCAGGGTCCGAGG-3’) was used as a reference RNA.

For qRT-PCR total RNA was extracted from the appropriately aged testes using RNeasy Mini Kit (Qiagen). Total RNA (500ng) was used for cDNA synthesis with oligo dT, Quantitative PCR was performed with SYBR green (Applied Biosystems, USA) on a Step One Real-Time PCR Systems from Bio-Rad according to the manufacturer’s instructions. Specific primers for qRT are the following: *upd* forward, 5’-GGGAGAGAGAGAGAAATAGAGAGA-3’ *upd* reverse, 5’-CGGGCGTGGCGAATAATA-3’; *Imp* forward, 5’-GCACCCACCACAATTTACAAC-3’, *Imp* reverse, 5’-CTCCAGGTTGCTTGCTTACT-3’; *DE-Cadherin* forward, 5’-CCGTAGACACTAAGACTCGATTAAG-3’, *DE-cadherin,* reverse, 5’-GTGTGGCTCTTCGTTTGTTG-3’; *rp49,* forward, 5’-CCAGTCGGATCGATATGCTAAG-3’, *rp49,* reverse, 5’-CCGATGTTGGGCATCAGATA-3’; *lin28*, forward 5’-CAGCCAGAGTTCCACTTCAT-3’, *lin28*, reverse 5’-GGCCACGTTGAACCATTTG-3’.

### Intensity measurements.

The intensity measurements for the samples were created in ImageJ (NIH) using RGB Profiler Macro. A rectangular selection around the hub cells and or individual cells, which were normalized with the background to get the accurate measurement. Similar settings were used for the all the measurement in the same day for the same set of experiments.

### Statistical analysis

All P values were calculated using a two-tailed unpaired t-test (GraphPad Prism 7) Average values are presented as mean ± s.e.m. Unless otherwise noted, *P<0.05, **P<0.01, ***P<0.001 and ****P<0.0001. n.s. not significant.

## SUPPLEMENTARY MATERIAL

Supplementary File
