# Supplementary material for: Lin28 is a critical factor in the function and aging of Drosophila testis stem cell niche
Source: Aging (Albany NY). 2019 Feb 4;11(3):855–73. doi: 10.18632/aging.101765 (PMC6382437; doi:10.18632/aging.101765)
Supplement: Supplementary File [file aging-11-101765-s001.pdf]

SUPPLEMENTARY MATERIAL

Supplementary Figures

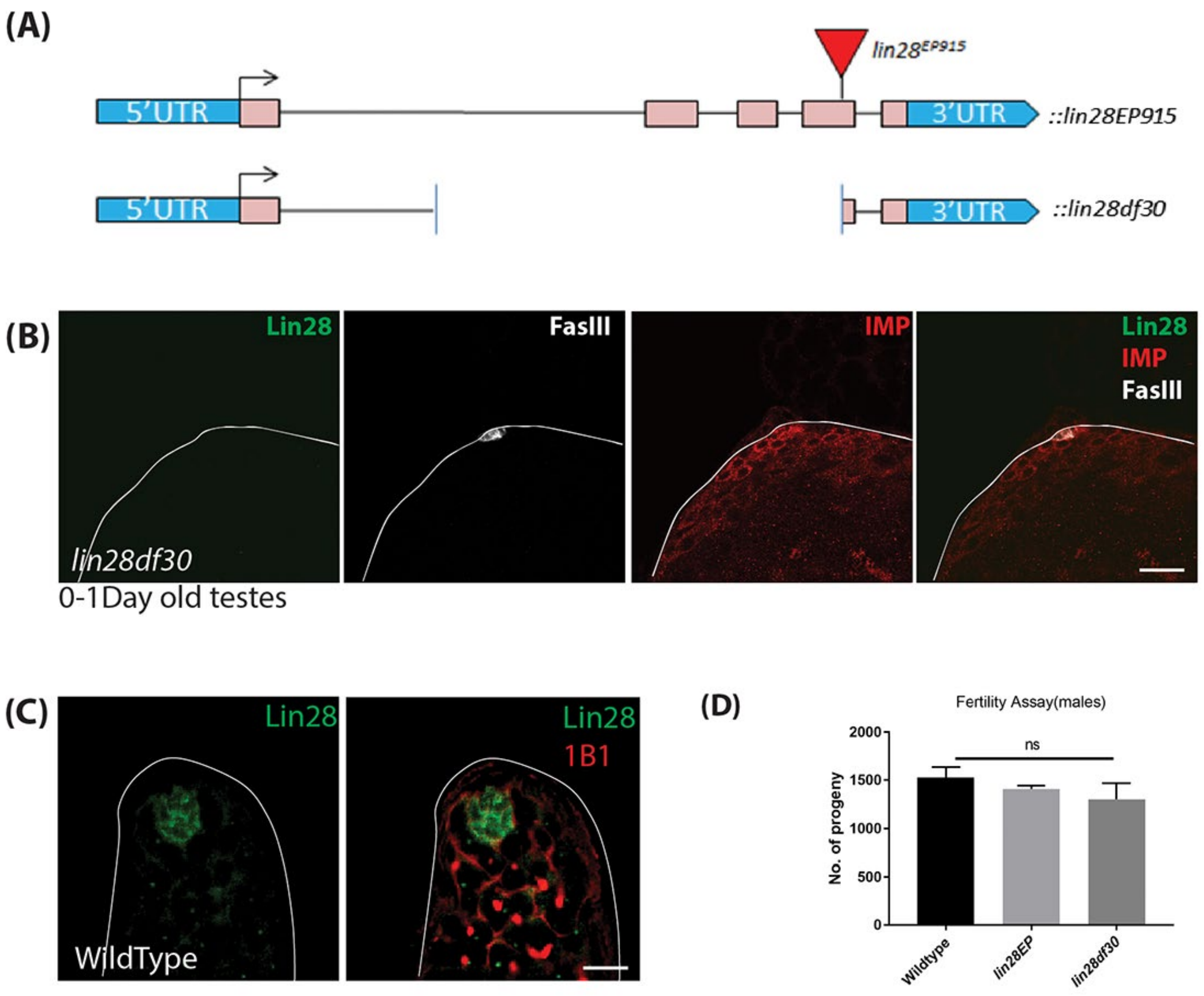

Supplementary Figure S1. Expression of Lin28. (**A**) A cartoon showing the P-element insertion site in the *lin28* gene locus along with the deletion mutant of *lin28*. (**B**) Expression of Lin28 in *lin28df30* showing complete loss of Lin28 expression (**C**). Expression of Lin28 in wildtype testes to show the specificty of antibody. (**D**) Fertility assay showing no significant change in number of progeny compared to WT vs mutants.Scale bar 10µm.

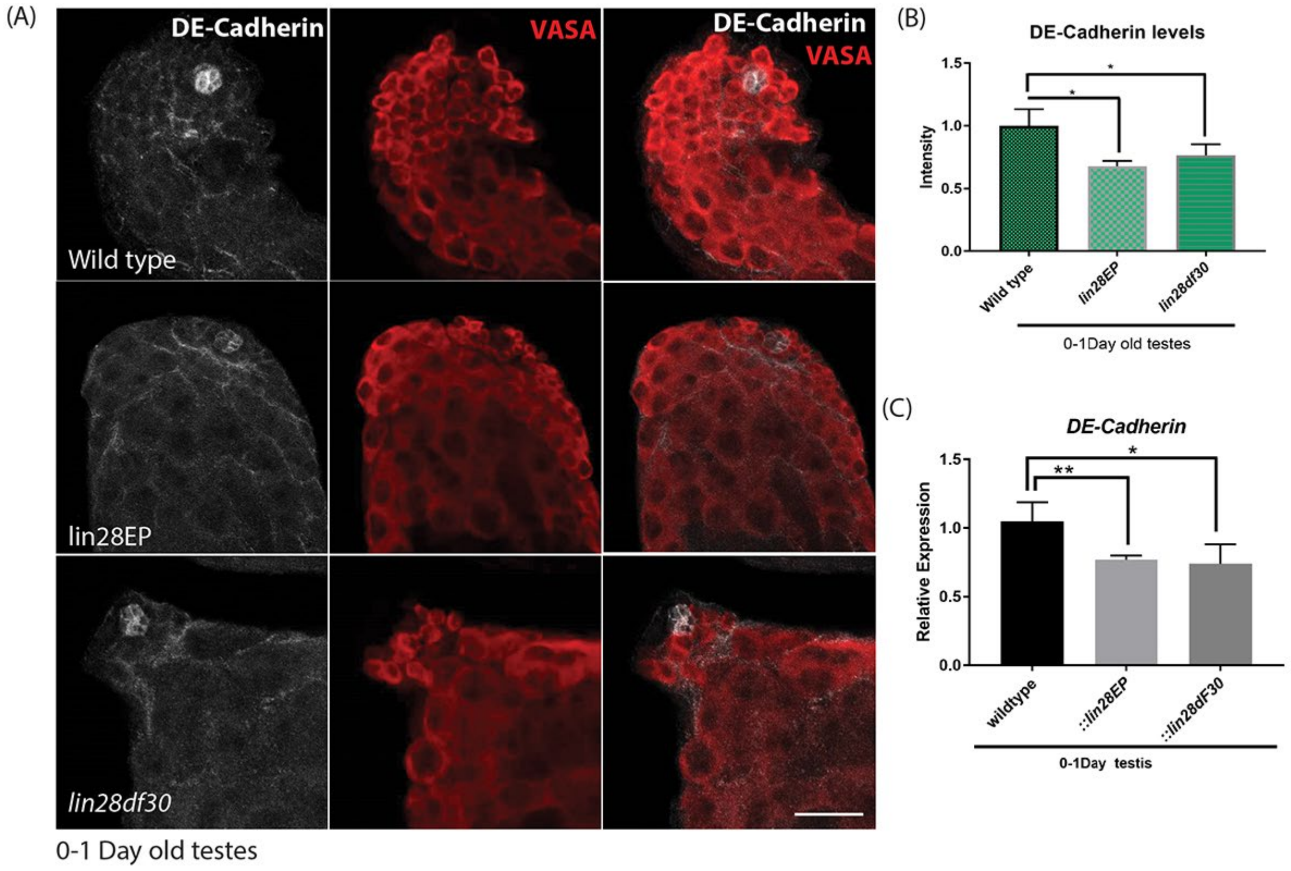

Supplementary Figure S2. Expression of DE-Cadherin. (**A**) Young testes stained with DE-Cadherin and VASA to show the loss in expression of DE-cadherin in the mutants. Scalebar 10µm. (**B**) Quantification of the intensity of DE-cadherin in hub area. WT n= 17; *lin28EP* n=22 ;l*in28df30* n=22. (**C**) RT-qPCR of young testes to show the reduction in *DE cadherin* in Lin28 mutant testes.

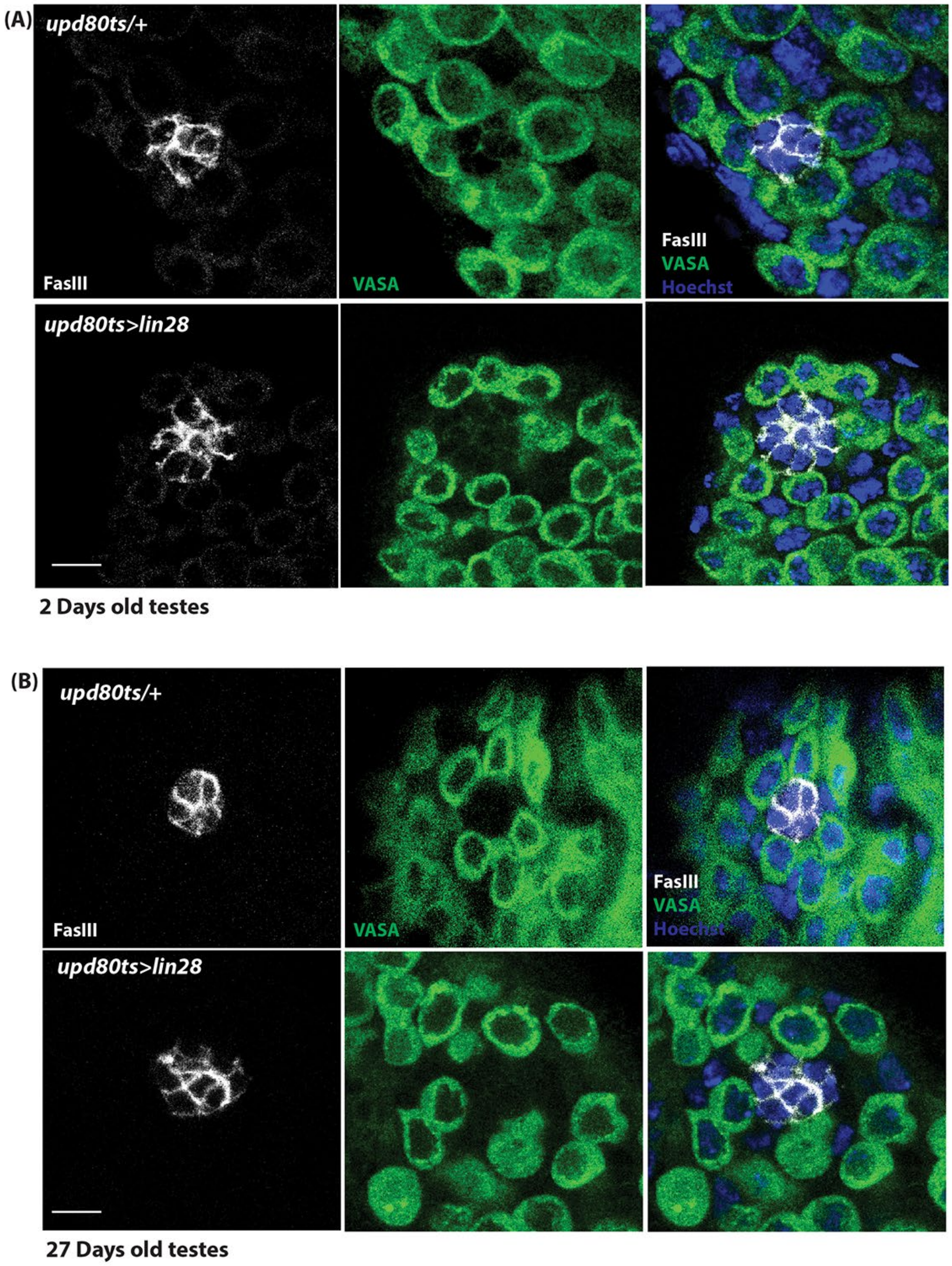

Supplementary Figure S3. Adult specific expressionof Lin28 in hub cells leads to increase in hub cell number. (**A**) 2 days after shifing to permissible temperature at 29CAdult testes stained with hub secific marker, FasIII and GSC marker VASA , shows increase in number of hub cells in overexpression of Lin28. (**B**) Testis stained after 27 days at 29°C shows Lin28 is required for maintaining the hub cell number in adult specific manner. Scale 10um.

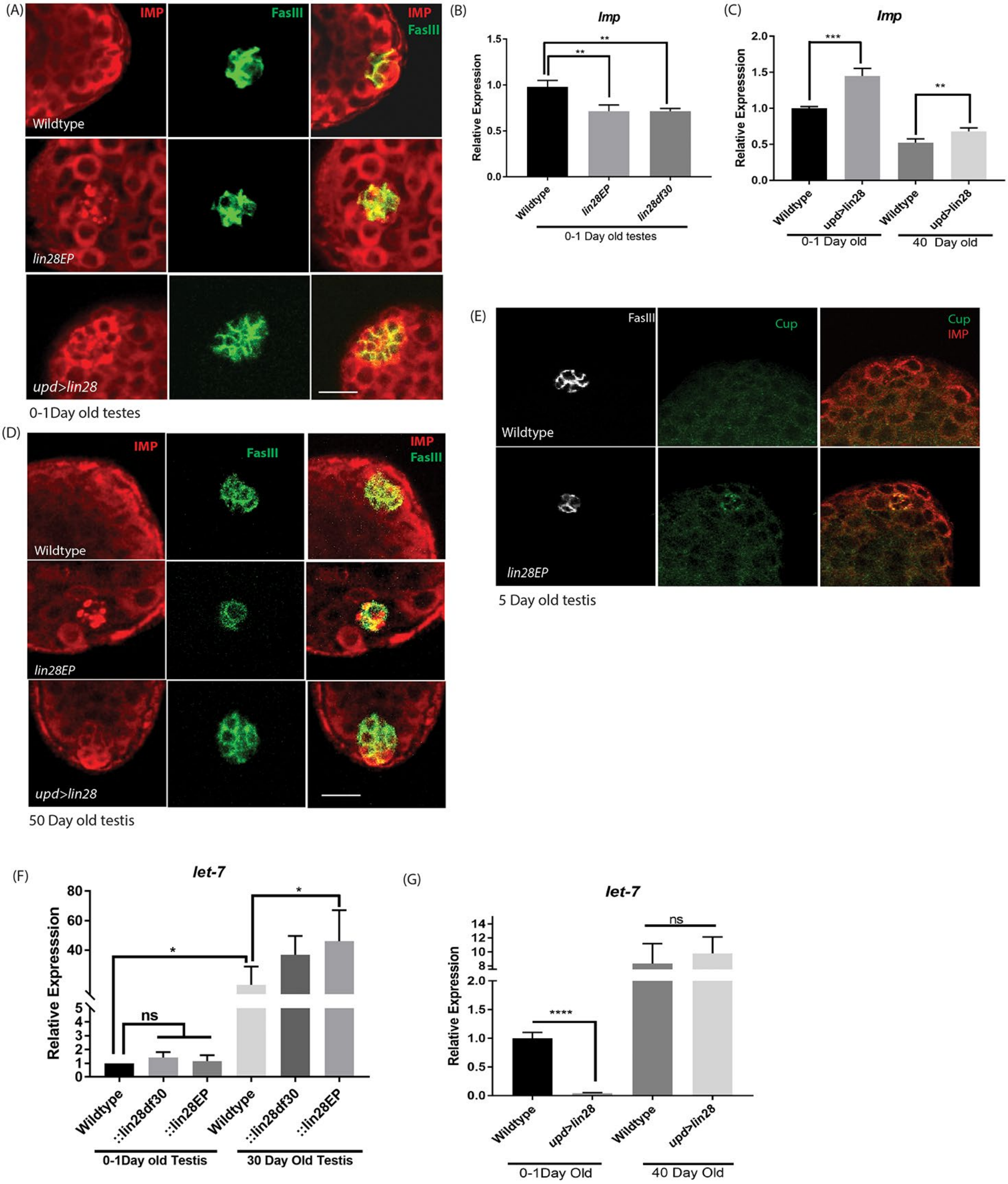

Supplemental Figure S4. Expression of IMP and let-7 does not play any role in the Lin28 mediated aging of hub cells. (**A**) Young testes stained with IMP antibody showing the accumulation of IMP into granular structures in hub are in lin28 mutant. (**B**) RT-qPCR of young testes showing loss of Imp transcripts in lin28 mutants. (**C**) qRT-PCR fo the expression of Imp transcripts in young and old testis, suggesting that over-expression of Lin28 leads to slight accumulation of Imp transcripts. (**D**) Old testes stained with IMP antibody showing the expression pattern of IMP in the hub cells. (**E**) 5 day old testes stained with Cup antibody along with IMP antibodies showing the co-localization of Cup and IMP into stress granules. (**F**) RT qPCR of young vs old testis to show that let-7 is not affected by Lin28 in the testes. (**G**) RT-qPCR of young and old testis suggesting no effect when Lin28 is overexpressed in old testes. Scale bar 10μm.

Supplementary Tables

Table S1. Quantification of hub cell number.

Genotype<br><br>**Young (0-1 Day)**	No. of Hub cells	No. of GSCs	No. of TJ positive cells (in apical hub region) excluding hub cells.	No. of testes analyzed
W1118	11.1 ± 0.2	9.05 ± 0.22	48.2 ± 0.44	N=55
:: lin28EP	6.53 ± 0.28	6.9 ± 0.25	34.7 ± 0.28	N=68
Upd-GAL4>UASlin28	13.6 ± 0.26	9.22 ± 0.27	44.8 ± 0.28	N=42
Upd-GAL4>UAS-lin28; lin28EP	10.3 ± 0.19	9.125 ± 0.26	-	N=17
**Old (50 Days)**				
W1118	7.6 ± 0.33	5.75 ± 0.29	22 ± 0.3	N=40
:: lin28EP	3 ± 0.30	3.3 ± 0.19	13 ± 0.28	N=60
Upd-GAL4>UASlin28	10.8 ± 0.36	7.4 ± 0.38	27.75 ± 0.32	N=38

Table S2. Quantification of Hub cell number.

Genotype<br><br>**Young (2 Days) @29ºC**	No. of Hub cells	No. of GSCs	No. of testes analyzed
upd-80ts/+	8.5 ± 0.33	7.8 ± 0.241	N=36
upd-80ts>UAS-lin28	11.43 ± 0.37	9.286 ± 0.28	N=42
**Old (27 Days) @29ºC**			
upd-80ts/+	5.68 ± 0.95	4.385 ± 0.85	N=39
upd-80ts>UAS-lin28	10.69 ± 0.26	8.33 ± 0.36	N=48
